# Quantification and modeling of turnover dynamics of *de novo* transcripts in *Drosophila melanogaster*

**DOI:** 10.1093/nar/gkad1079

**Published:** 2023-11-24

**Authors:** Anna Grandchamp, Peter Czuppon, Erich Bornberg-Bauer

**Affiliations:** Institute for Evolution and Biodiversity, University of Münster, Münster, Germany; Institute for Evolution and Biodiversity, University of Münster, Münster, Germany; Institute for Evolution and Biodiversity, University of Münster, Münster, Germany; Department of Protein Evolution, Max Planck Institute for Biology, Tübingen, Germany

## Abstract

Most of the transcribed eukaryotic genomes are composed of non-coding transcripts. Among these transcripts, some are newly transcribed when compared to outgroups and are referred to as *de novo* transcripts. *De novo* transcripts have been shown to play a major role in genomic innovations. However, little is known about the rates at which *de novo* transcripts are gained and lost in individuals of the same species. Here, we address this gap and estimate the *de novo* transcript turnover rate with an evolutionary model. We use DNA long reads and RNA short reads from seven geographically remote samples of inbred individuals of *Drosophila melanogaster* to detect *de novo* transcripts that are gained on a short evolutionary time scale. Overall, each sampled individual contains around 2500 unspliced *de novo* transcripts, with most of them being sample specific. We estimate that around 0.15 transcripts are gained per year, and that each gained transcript is lost at a rate around 5× 10^−5^ per year. This high turnover of transcripts suggests frequent exploration of new genomic sequences within species. These rate estimates are essential to comprehend the process and timescale of *de novo* gene birth.

## Graphical Abstract

**Graphical Abstract:**
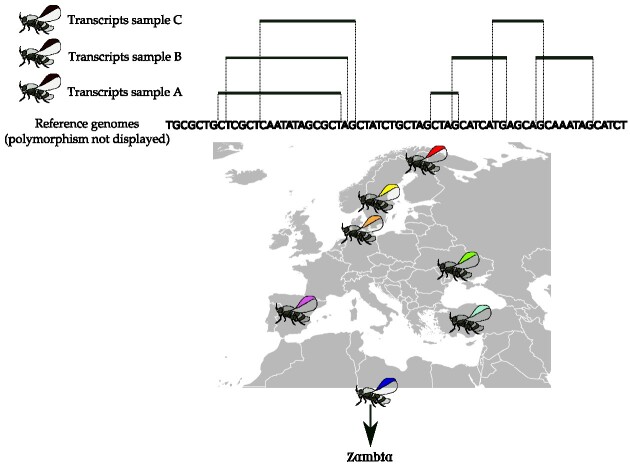

## Introduction

In most multicellular organisms, only a small fraction of the genome codes for proteins (1,2). Intriguingly though, a large fraction of the non-genic genome is transcribed too, at least occasionally, e.g. under specific conditions such as stress or during development (3–5). This production of transcripts throughout the entire genome has been described as pervasive transcription (6–8) and has been demonstrated through several techniques (reviewed in (9)). Many non-genic transcripts perform important functions. For example, rRNA and tRNA are indispensable constituents for the protein assembly by the ribosome (10). Some long non-coding RNAs (transcripts with >200 nucleotides) are involved in the regulation of splicing (11) and transcription in general (12–15). Among non-coding transcripts, some are found only in a single species or population without a known ortholog and can therefore be defined as *de novo* transcripts.

Interestingly, many *de novo* transcripts were found to bind to ribosomes, indicating that part of their sequence might contain *Open Reading Frames* (ORFs) that are likely translated into small proteins (16–21). This protein-coding potential of *de novo* transcripts makes them important precursors of *de novo* genes, which are coding genes that arise from previously non-coding genomic regions (22). Together with the gain of an ORF, the gain of transcription is a fundamental feature of *de novo* gene emergence (23–27). To understand the evolution and assess the rate of *de novo* gene emergence, it is therefore critical to quantify the evolutionary dynamics of *de novo* transcripts (26,28–30).

The rate at which *de novo* transcripts are gained and lost is still largely unknown. Studies across multiple species indicate high turnover, i.e. gain or loss, of long non-coding RNAs (reviewed in (31)). For example, Necsulea *et al.* (32) estimate that around 10 000 *de novo* transcripts emerged and were fixed in primates since the split from rodents around 100 million years (MY) ago, indicating a fixation rate of 100 transcripts per MY. A similar analysis, based on a comparison of transcriptomes from several rodent species, found a fixation rate of 5–10 transcripts per MY (33). In these studies, transcripts were compared between species separated by large phylogenetic distances. This provides important insight into the transcript turnover in conserved genomic regions. However, a large amount of transcripts could not be compared because conserved genomic regions only cover a small fraction of the genome due to genome evolution and rearrangements. Moreover, the total turnover of transcripts is estimated without an underlying evolutionary model of transcript gain and loss. For example, the difference between the number of transcripts in rat and mouse genomes can be either due to a loss in the mouse species or a gain (and subsequent fixation) in the rat species, which is impossible to distinguish with the existing data (33).

Here, we aim to overcome these limitations by using a tightly controlled setting and very short phylogenetic distances. We use deep sequencing data from seven genomes of individual samples from a single species: *Drosophila melanogaster*. These genomes were extracted from inbred lines from different geographic locations. Because we used samples from an almost panmictic population from the same species, transcriptome comparisons are more precise than for samples coming from different species. We assembled a genome and a transcriptome for each homozygote DNA and RNA of the inbred lines, and used comparative genomics approaches to detect newly emerged transcripts in each sample. We additionally determined the genomic location of the *de novo* transcripts and determined orthology with newly proposed transcript-specific orthogroup definitions. The occurrence of transcripts in different samples allows us to infer transient dynamics of transcripts, i.e. their gain and loss rates. We call the birth of a transcript a *gain*, which importantly does not imply its fixation in the population. It may well be, and in fact is much more likely, that a newly gained transcript will be lost again before it is fixed in the population or species. Transient gain and loss rates were estimated using the infinitely many genes model (34). To test the robustness of our results, we also applied a model that accounts for gene flow between the samples, and a model that takes specific phylogenetic information about the samples into account (details in the Supplementary Information (SI)). In contrast to previous studies, these estimates of gain and loss account for the evolutionary turnover of transcripts between samples of a single species, instead of solely the fixation (or loss) between different species. In addition to these transient processes, we also estimate the number of transcripts that have become fixed in our sample, in comparison to close Dipteran outgroups. Our study therefore provides a detailed picture of transcript turnover rates and evolutionary dynamics, which is important information to understand the process of *de novo* gene birth.

## Materials and methods

### Sample-specific reference genomes

RNA short reads from whole genome illumina sequencing and DNA long reads from whole genome nanopore sequencing of seven isofemale lines of *D. melanogaster* were downloaded from NCBI (accession PRJNA929424). Among the seven lines, six come from Europe (Finland (FI), Sweden (SE), Denmark (DK), Spain (ES), Ukraine (UA) and Turkey (TR)) and one is from Zambia (ZI) (SI, Section A.1). The RNA-Seq samples were built based on total RNA from two males, two females and one larva pooled together (35). The DNA was extracted from 50 individuals per inbred line. For each inbred line, we refer to the genetic material that was extracted and assembled from the line by a *sample*. The sample from Zambia is considered as the ancestral outgroup as the *Drosophila melanogaster* from sub-Saharan Africa diverged from the European populations around 13 000 years ago (36,37).

Our model and pipeline was set up to assess transcript gain and loss within a species. Reference genomes were assembled for each sample by mapping the long DNA read of the sample to a reference genome of *D. melanogaster*, and extracting the seven consensus genomes. This methodology has two advantages. First, it allows to compare the precise location of transcripts between each sample, and indeed allows to use the genome annotation to compare transcription between samples. It also made possible for us to build three definitions of transcript orthology with more comparable genomic location than from *de novo* assemblies. Second, as most studies of population genomics only use one reference genome for a species, our method can directly be applied in this context. However, the choice of not using the *de novo* assembled genomes also has a cost. We suspect that part of the transcript assemblies have been lost, e.g. due to new putative TE insertions, genome duplications or inversions that can be found frequently in *Drosophila*. After several control steps, we estimate a maximum of 130 missed unspliced *de novo* transcripts (average number of transcripts of 28,021; SI Sections A.4, A.5 and A.10 Supplementary Figures S9 and S10). For each sample, DNA reads shorter than 100 bp were removed from the long DNA reads by using Filtlong (github rrwick/Filtlong). We used the genome of *D. melanogaster* BDGP6.28, downloaded from Ensembl (38), as a reference genome. DNA long reads from each sample were mapped to the reference genome using BWA-MEM (39) and a consensus genome per sample was extracted. The resulting SAM files were converted into BAM with samtools-view (40). The BAM files were sorted and indexed with samtools-sort and samtools-index. Mapping statistics were obtained with samtools-flagstats (SI, Section A.2), and alignments were visualized with *Integrative Genome Viewer* (41). This procedure mapped 95–98% of the DNA to the reference genome. For each sample, a consensus genome was extracted, by using samtools-mpileup (42), bcftools (43) and its function vcfutils.pl (44) (Supplemental Deposit). The genomic regions that were not covered by mapping were completed by the corresponding region of the reference genome of *D. melanogaster*. Percentages of polymorphisms were assessed between samples, as well as genomic GC contents (SI, Section A.3). The nucleotide polymorphism between samples was systematically lower than the threshold of 2%, confirming that the DNA belongs to a single species. This approach allowed us to detect genomic polymorphisms between samples, and to increase the precision for mapping sample-specific transcripts to genomes.

### Transcriptome assembly

RNA reads were trimmed with Trimmomatic (0.32) to remove the adaptors AGATCGGAAGAGCACACGTCTGAACTCCAGTCA in forward and AGATCGGAAGAGC GTCGTGTAGGGAAAGAGTGT in reverse (45). The quality of reads was assessed with FastQC (0.11.9) (46). The seven reference genomes were indexed with HISAT2 (47), and RNA reads from each sample were mapped to their respective reference genome with HISAT2 using the spliced aware option. The resulting SAM files were converted to BAM format with SAMtools (40). The BAM files were sorted and indexed. In each sample, the transcriptomes were assembled with StringTie (48) (Supplemental Deposit). The GTF files of the assembled transcriptomes were converted into FASTA with the FASTA2GTF module from gffRead (49). Additionally, GFF3 files of the assemblies were generated with the script ‘gtf_to_alignment_gff3.pl’ from TransDecoder/Trinity (50) (Supplemental Deposit). For each sample, a final file was generated including the genomic position of unspliced transcripts, transcription orientation, and the size of the unspliced transcripts. Transcript coverage was recorded as *Transcripts Per Million* (TPM). The GTF file of the reference *D. melanogaster* genome was retrieved to access the position of established genes and transposable elements, which were annotated in the seven new reference genomes.

We estimated if the use of genomes assembled by mapping resulted in loss of *de novo* transcripts compared to *de novo* assembly. The unmapped transcripts were retrieved in each sample with bedtools and converted into FASTA. The unmapped transcripts were used as a query for a nucleotide BLAST search against a database of annotated TEs for insects from RepeatMasker (51). Moreover, the transcriptome assemblies generated with *de novo* genome assemblies were retrieved from (35), and used as a BLAST query against the transcriptomes generated in this manuscript. We determined the genomic positions of transcripts without hits, and the ones corresponding to normal gene expression were annotated with bedtools.

### Detection of *de novo* transcripts

The transcriptomes of six *Drosophila* species (*Drosophila simulans, Drosophila sechellia, Drosophila virilis, Drosophila ananassae, Drosophila yakuba, Drosophila erecta*) were downloaded from Ensembl metazoa (http://metazoa.ensembl.org/index.html). These transcriptomes included all known protein-coding transcripts extracted from male and female *Drosophila*, as well as predicted coding gene transcripts. We also downloaded the whole set of annotated non-coding RNAs referenced for these species. To weed out as many transcripts of older origin as possible, these transcriptomes were merged and used as a target for nucleotide BLAST search (52) with all sample-specific transcripts as query. Nucleotide BLASTs were performed with a cutoff of *E* = 10^−2^, in the forward direction of the target transcripts. Transcripts with no hit were considered as preliminary *de novo* transcripts. Preliminary *de novo* transcripts were then used as query to BLASTn search against the transcriptome of seven other Dipteran species: *Aedes aegypti, Anopheles arabiensis, Culex quiquefasciatus, Lucilia cuprina, Musca domestica, Stomoxys calcitrans, Teleopsis dalmanni*, downloaded from Ensembl, with a cutoff of *E* = 10^−2^, in the forward direction of the target transcripts. The remaining *de novo* transcripts without a BLAST hit were then filtered by TPM value. Transcripts with a TPM <0.5 were removed from the data set to exclude low frequency transcripts. The remaining transcripts were considered as *de novo* transcripts (Supplemental Deposit). The same analysis was performed again with higher thresholds of expression of 1 and 5 TPM (data in SI, Section A.7). All of the follow-up analyses in the main text were performed on the *de novo* transcripts detected with a threshold of 0.5 TPM (TPM 1 and 5 analysis results are provided in SI, Section B.4).

The genomic positions of unspliced transcripts were retrieved from the seven GTF files generated from the transcriptome assemblies, and the overlap with genomic components was assessed with bedtools (53) and a python code developed for this purpose. The transcripts were distributed in six genomic positions: (a) ‘Overlapping with an intergenic region and a fragment of a gene in the identical direction’, referred to as ‘exon longer’, (b) ‘Overlapping with an intergenic region only’, referred to as ‘intergenic’, (c) ‘Overlapping with an intergenic region and a fragment of a gene but in the opposite direction’, referred to as ‘antisense’, (d) ‘Overlapping with an intergenic region and a pseudogene’, referred to as ‘pseudogenic’, (e) ‘Overlapping with an intergenic region and an annotated non-coding RNA’, referred to as ‘ncRNA’ and (f) ‘Inside of an intron’ referred as ‘intronic’.

### Orthogroups of *de novo* transcripts

*De novo* transcripts from the seven samples were searched for orthology relationships between them. Constructing orthogroups of transcripts is more complex than constructing orthogroups of protein-coding genes. Two protein-coding genes are commonly grouped together into an orthogroup if the sequence similarity and coverage of their encoded protein exceeds a certain threshold, which suggests a common origin and a homologous function of the encoded protein. On the other hand, two transcripts can overlap but have arisen from a completely different transcription process if their transcription initiation and termination sites do not coincide. Moreover, two transcripts can have similar initiation and termination sites, but are differently spliced in different samples, giving rise to spliced transcripts with low sequence homology. Indeed, depending on the scientific question, transcript homology should be defined differently.

**Figure 1.**
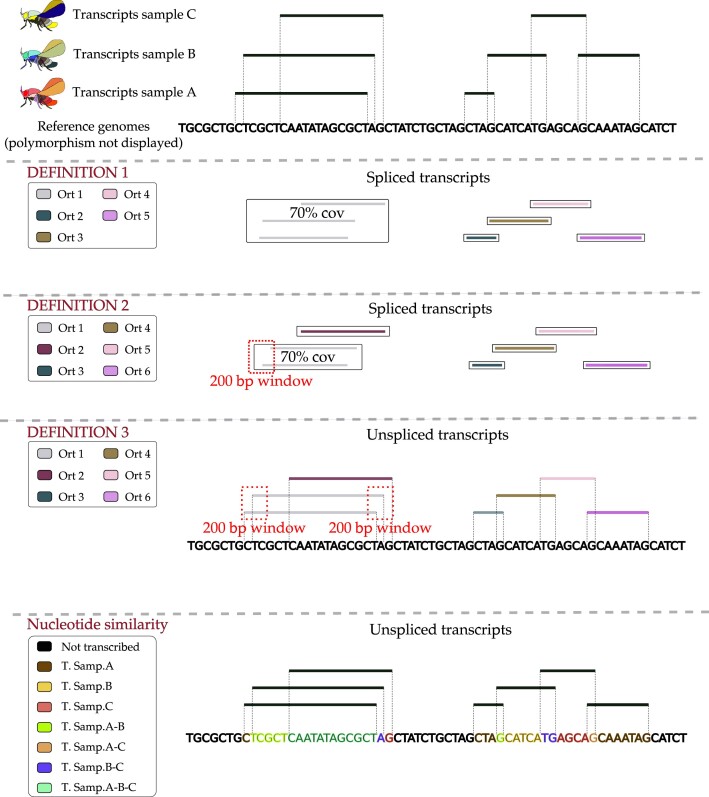
Transcript classifications. The figure illustrates the different *de novo* transcript definitions and the additional approach to categorize transcript nucleotides. Three samples are shown as an example. The black nucleotides represent the reference genomes (all of the exact same size), the colored lines at the top show the transcripts.

To cover a large number of possible scenario, we established three different definitions of transcript orthology that are depicted in Figure 1:

Definition 1: A set of transcripts are considered orthologous if their spliced sequences share at least 70% of coverage between all of the members of the orthogroup, and 75% of identity in reciprocal BLASTn.
Definition 2: A set of transcripts are considered orthologous if their spliced sequences share at least 70% of coverage between all of the members of the orthogroup and 75% of identity in reciprocal BLASTn, and the transcription initiation sites of all of them can be found in a window of 500 bp of the genome.
Definition 3: A set of transcripts are considered orthologous if their unspliced sequences start at a genomic position that is within a window of 500 bp, and end in a window of 500 bp.

*Definitions 1* and *2* compare spliced transcripts. *Definition 2*, in addition to sequence similarity, takes into account the genomic position of the transcription initiation site, thus making orthogroup assignment more restrictive than *Definition 1*. In contrast, *Definition 3* builds on the initiation and termination sites but does not consider sequence similarity. In this definition, two splice variants of a single transcript would be clustered together, independently of the sequence similarity of the spliced variants. Therefore, if two transcripts emerge at the same position in two samples but with different splicing variants, they would still be detected by this definition.

To build the orthogroups following *Definitions 1* and *2*, *de novo* transcripts from each sample were used as target for BLASTn searches against the *de novo* transcripts of the six other samples. With a 70% coverage threshold, we did not encounter any ambiguous orthogroup definitions, e.g. when multiple transcripts overlapped with 70%, but not for all pairwise comparisons. The choice of 70% coverage is to some extent arbitrary. As a control, the same data were extracted with a coverage threshold of 30% for *Definition 1* and 90% for *Definitions 1* and *2*. The choice of threshold did not affect the results (SI, Section B.5, data provided in the Supplemental Deposit). A python script was used to sort the orthogroups according to our definitions. For *Definition 3*, a script was built to directly assess the overlapping positions and nucleotides of *de novo* transcripts between samples. To test the accuracy of our definitions, we used a set of 11 000 proto-genes from Grandchamp *et al.* (54), and assigned them to orthogroups by using the software OrthoFinder (55) and our script for *Definition 1*. We found 93% of similarity between the results from OrthoFinder and our script (OrthoFinder: 5687 orthogroups, our script: 6124 orthogroups) (Supplemental Deposit). The difference of 7% is due to a higher coverage threshold in our pipeline compared to OrthoFinder.

Additionally, we used an alternative approach to estimate the similarity of transcripts between the samples. Instead of comparing transcripts with each other and treating them as a single object, we compare transcribed nucleotides. For example, if two transcripts of 200 bp found in two samples have an overlap of 50 bp, we will consider these 50 nucleotides as ‘*de novo* transcribed nucleotides’ common to the two samples, and the remaining 150 nucleotides as ‘*de novo* transcribed nucleotides’ specific to each sample. We used a python script to classify the nucleotides of transcripts into these categories. We refer to this approach as ‘Nucleotide similarity’.

### Evolutionary model of transient dynamics of *de novo* transcripts

We estimate gain and loss rates of *de novo* transcripts using a model that describes transcript gain and loss dynamics along the ancestry of the *D. melanogaster* samples. This evolutionary model is based on the *infinitely many genes model*, which has been developed to describe the gain and loss dynamics of genes in prokaryotes (34,56). Analogously to the infinitely many genes model, in our model each *de novo* transcript arises only once during the short evolutionary time frame that we study. This means that any *de novo* transcript that is shared between different samples must have been gained in one of the ancestral branches common to both samples. Furthermore, in this model *de novo* transcripts are selectively neutral, i.e. they do not confer a fitness advantage or disadvantage, which is in line with empirical evidence (57).

*De novo* transcripts are gained with probability *u* per unit of time and each transcript is lost with probability *v* per unit of time. Here, we use a generation as a unit of time. To translate between generations and years, we assume that the generation time of *D. melanogaster* is approximately 2 weeks (58). The total number of *de novo* transcripts in a sample after *t* units of time, denoted by *g*(*t*), is then described by the following dynamics (e.g. Eqs. (6), (7) in (59)):


(1) \begin{equation*} \frac{dg(t)}{dt} = u - v g(t) \qquad \Rightarrow \qquad g(t) = \frac{u}{v}\left(1-e^{-vt}\right)\, . \end{equation*}

The equilibrium number of *de novo* transcripts therefore is *u*/*v*.

### Estimating *de novo* transcript gain and loss rates

Next, we outline how to estimate the gain and loss rates of *de novo* transcripts. To this end, we compare the empirical transcript frequency spectrum to a theoretical prediction of the frequency spectrum. The transcript frequency spectrum contains information about the number of transcripts shared by a certain number of samples. We denote by $D^n = (d_1^n,...,d_n^n)$ the empirical transcript frequency spectrum and by $T^n=(t_1^n,...,t_n^n)$ the theoretical prediction for the transcript frequency spectrum, where *n* is the number of samples that are studied. In the main text we restrict the study to the European samples, i.e. *n* = 6, whereas in Section B.3 in the SI we also include the Zambian sample in the analysis, i.e. *n* = 7. To estimate the parameters, we use a χ^2^ statistic to compare the empirical and theoretical frequency spectra, as has been done before (56,59):


(2) \begin{equation*} \chi ^2 = \sum _{k=1}^n \frac{(d^n_k - t^n_k)^2}{t^n_k} . \end{equation*}

We now outline how to compute the theoretical transcript frequency spectrum following Baumdicker *et al.* (34,56). The genealogy of the samples is modeled by a standard coalescent. The coalescent describes the ancestral relationship of samples taken from a neutrally evolving population of individuals in an unstructured population (60). In this setting, the genealogy of the sample is given by a standard coalescent and the theoretical frequency spectrum can be computed analytically (34). The European (meta-)population of *D. melanogaster*, despite showing some partition into a heterogeneous Western and a homogeneous Eastern cluster (61), overall has only a relatively weak population structure (*F*_*ST*_ values of 0.01–0.06 as estimated in Kapun *et al.* (62)). In view of these previous results, the genealogy of the European samples is then reasonably well described by a standard coalescent. We therefore restrict our analysis in the main text to these samples. The extended data set including the Zambian sample is analyzed in Section B.3 in the SI and shows no substantial difference in the estimated rates per generation (Table B.9 in the SI). Additionally, to study the robustness of the parameter estimation using coalescent theory, we also explore a model where the ancestry is given by the estimated phylogeny of the sample (59) (SI, Section B.1 and Section A9 Supplementary Figures S2–S8). Using this alternative approach to estimate gain and loss rates, we do not find substantial differences to the reported estimates below (details in the SI, Sections B.1 and B.3).

**Table 1.** Number of identified transcripts and *de novo* transcripts in the analysed samples

Samples	DK	ES	FI	SE	TR	UA	ZI
	(Denmark)	(Spain)	(Finland)	(Sweden)	(Turkey)	(Ukraine)	(Zambia)
# transcripts	29 675	27 901	28 212	27 022	27 357	27 786	28 198
# *de novo* transcripts	2908	2842	2708	2714	2997	3024	3116
# *de novo* unspliced transcripts	2344	2417	2327	2320	2529	2620	2809

**Table 2.** Number and proportion of *de novo* transcripts per sample ordered by genomic region

Position	DK	ES	FI	SE	TR	UA	ZI
Exon longer	307	355	352	351	362	366	396
	10.5%	12.5%	13%	13%	12%	12%	12.5%
Intergenic	577	571	537	415	664	731	707
	20%	20%	20%	15.5%	22%	24%	22.5%
Intronic	63	79	69	59	61	106	67
	2%	3%	2.5%	2%	2%	3.5%	2%
ncRNA	108	111	140	115	131	112	210
	3.5%	4%	5%	4%	4.5%	4%	7%
Pseudogene	11	12	8	10	13	10	12
	0.5%	0.5%	0.5%	0.5%	0.5%	0.5%	0.5%
Antisense	1842	1714	1602	1764	1766	1699	1724
	63.5%	60%	59%	65%	59%	56%	55.5%

**Table 3.** Estimated *de novo* transcript gain and loss rates. The gain and loss rates are measured as rates per year, the parameter *C*_fixed_ estimates the number of fixed transcripts in the sample

Model	Gain	Loss	Gain (slow)	Loss (slow)	*C*_fixed_
**Definition 2**					
1 transcript class	0.13	5 × 10^−5^	–	–	91
2 transcript classes	0.15	2 × 10^−4^	0.06	3.1 × 10^−5^	50
**Definition 3**					
1 transcript class	0.17	6.3 × 10^−5^	–	–	95
2 transcript classes	0.28	2.6 × 10^−4^	0.05	3 × 10^−5^	52

The frequency spectrum is given by gain and loss rates per generation. To transform these estimates to the time scale of the coalescent, we write θ = 2*N*_*e*_*u* and ρ = 2*N*_*e*_*v*, where *N*_*e*_ denotes the effective population size. In our specific setting, *N*_*e*_ corresponds to the effective population size of the European *D. melanogaster* population, here set to *N*_*e*_ = 900 000 as estimated in Laurent et al. (37). Then θ is the average number of gained transcripts in 2*N*_*e*_ generations and ρ is the rate at which a transcript is lost in 2*N*_*e*_ generations. To transform these parameters to the scale of years, the estimated values are multiplied with the factor: *no. of generations per year*/(2× *effective population size*), where we assume 26 generations per year, which is based on a generation time of two weeks (58).

We denote by $G_k^n$ the number of transcripts shared by *k* samples out of the *n* samples. Similar to Collins and Higgs (59), we also study two transcript classes: transcripts with high and low turnover. To distinguish their respective rates, we write θ_*s*_, ρ_*s*_ for the ‘slow’ class and θ_*f*_, ρ_*f*_ for the ‘fast’ class of transcripts. We drop the indices if we use only one class of transcripts in the following. The expected frequency spectrum, denoted by $\mathbb {E}[\cdot ]$, in its most general form is then given by ((34), Theorem 5 extended to multiple classes)


(3) \begin{eqnarray*} \mathbb {E}\left[G_k^n\right] &=& \sum _{i\in \lbrace s,f\rbrace } \frac{\theta _i}{k}\ \frac{n \cdots (n-k+1)}{(n-1+\rho _i)\cdots (n-k+\rho _i)}\, , \text{ for } 1\le k \,<\, n\, ,\nonumber\\ && \mathbb {E}\left[G^n_n\right] = \sum _{i\in \lbrace s,f\rbrace } \frac{\theta _i}{n}\ \frac{n \cdots 1}{(n-1+\rho _i)\cdots \rho _i} + C_{\text{fixed}}\, , \end{eqnarray*}

where *C*_fixed_ denotes the number of *de novo* transcripts that are fixed in our sample, which means that they have a loss rate equal to zero and are found in all samples.

#### Numerical implementation of the parameter estimation

We used python and the integrated ‘minimize’ function from SciPy specifying the method ‘SLSQP’ to obtain parameter estimates through Eq. (2). We estimated parameters using one or two classes of transcripts plus the number of fixed transcripts, i.e. we estimated either three or five parameters. We constrained the parameters so that the mean number of transcripts per sample, the value *u*/*v* or θ/ρ, fits the empirical observation $\sum _{i=1}^7 i d_i^n / n$. We did this to improve convergence of the minimization procedure by reducing the number of parameters to be estimated, which was necessary in the multi-class case. In addition, we conditioned the loss parameter(s) to be between 0 and 1000 and the gain parameter(s) to be between 0 and 20 000.

Initial values for the parameters in the optimization routine were chosen by fitting the mean number of transcripts per sample and the pairwise sample differences if we fitted one class of transcripts, as reasoned in Baumdicker *et al.* (56) (more details in the SI, Section B.2). When fitting two classes of transcripts we divided the initial guess of the gain rate by two and set the initial gain and loss rate estimates of the slow transcript class to the initial values of the fast class divided by 100. All choices of initial parameter values are stated in Section B.2 in the SI.

### Programming and analyses

All statistical analyses were performed with R (63). Data processing, analyses, orthology searches and modeling were performed with python (64), and can be accessed at: https://github.com/AnnaGrBio/Transcripts-gain-and-loss.

## Results

### Sample-specific genome and transcriptome assemblies

The seven samples were extracted from seven inbred isolines of *Drosophila melanogaster* from different geographic locations (six from Europe, one from Zambia; details are provided in the Methods and Supplementary Information (SI), Section A.1). To this end, we compiled sample-specific reference genomes and identified *de novo* transcripts.

We mapped long DNA reads of each sample to the reference genome of *D. melanogaster* and extracted the seven consensus genomes. The percentage of DNA reads that correctly mapped to the reference genome ranged from 94.3% to 97.42% (SI, Section A.2 and Supplemental Deposit: https://doi.org/10.5281/zenodo.7681079). The percentages of SNPs between the aligned genomes of the seven samples ranged from 0.22% to 0.58% (SI, Section A.3 Supplementary Figure S1), showing very low divergence as expected for samples of the same species. The Zambian sample diverged most from the other samples, which is consistent with its geographic separation from the European samples.

### *De novo* transcripts in samples

RNA reads from each of the seven samples were then mapped to their respective reference genome to build sample-specific transcriptomes (Supplemental Deposit; SI, Section A.2). *De novo* transcripts were identified in the seven samples of *D. melanogaster* (details in the Methods section). In total, an average of 28,021 transcripts were found per sample (Table 1). Among these, on average 2,901 transcripts per sample were identified as *de novo* transcripts, as they showed no detectable homology to transcripts in other Diptera species, and had a required minimal expression threshold of 0.5 *Transcripts Per Million* (TPM) (Table 1). Among these transcripts, some arose via alternative splicing from a single unspliced precursor. When merging splicing variants as a single transcript, the average number of *de novo* transcripts dropped by 14.5% to an average of 2481 per sample (Table 1). *De novo* transcripts were also defined with higher thresholds of expression. For example, when using 1 TPM as a minimum level of expression of *de novo* transcripts, on average 871 transcripts were removed per transcriptome (SI, Section A.6), amounting to a total of 2030 transcripts per sample. Genome assembly by DNA mapping can introduce a bias while assembling transcriptomes as events of sample-specific indels and TEs are missing. Putative bias due to TE insertions and transcript losses compared to *de novo* genome assembly were assessed (SI, Sections A.4 and A.5). We estimated that an average of 130 spliced (60 unspliced) *de novo* transcripts were lost in the assembly.

*De novo* transcripts were distributed uniformly across the chromosomes where they emerged (SI, Section A.3) and were found in all chromosomes except the mitochondrial one. According to their overlap with annotated genomic elements, we defined six different genomic positions to characterize the transcripts (details in the Methods section). Interestingly, *de novo* transcript distribution followed a similar pattern in all seven samples (Table 2): Around 60% of *de novo* transcripts overlapped with annotated genes in antisense, around 20% of the transcripts were found to be entirely intergenic, and just very few transcripts (around 3%) emerged inside introns. Except for these intronic transcripts, transcripts from all other genomic positions also overlapped, at least partially, with intergenic regions, making the intergenic region a very important pool of putative emergence of transcription initiation and termination. In addition, 8–12 *de novo* transcripts were consistently found to overlap with annotated pseudogenes in each sample. These pseudogenic transcripts were exceptionally long (11 000–150 000 nucleotides, Supplemental Deposit). Transcripts were also observed to overlap with non-coding RNA (ncRNA), but with an initiation site upstream or a termination site downstream of it. Lastly, we found that *de novo* transcripts contain an average of 0.37 introns (SI, Section A.7 Supplementary Table A.1).

### Orthogroups of *de novo* transcripts

**Figure 2.**
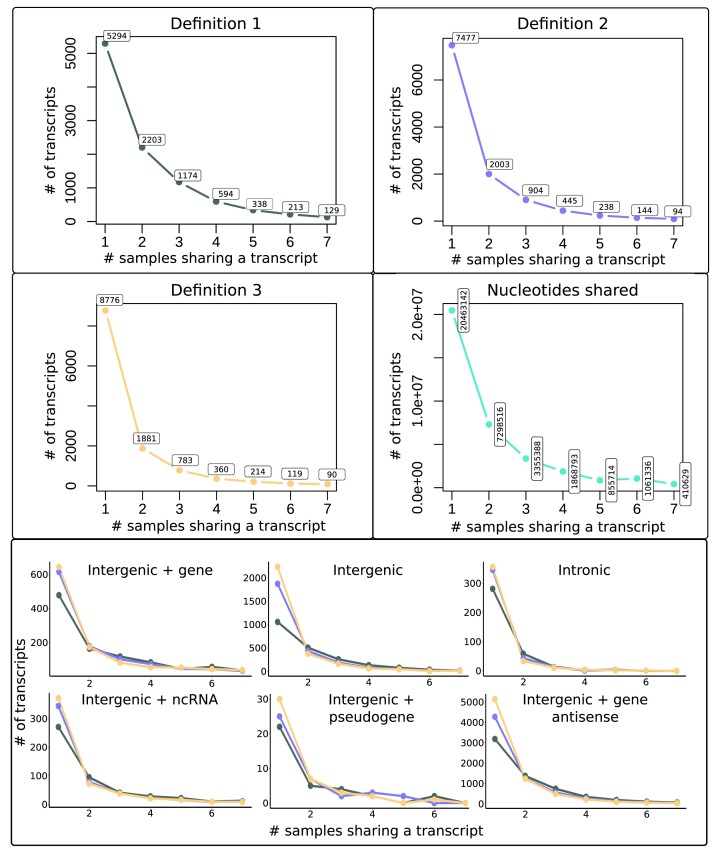
*De novo* transcripts shared by samples. The graphs from *Definitions 1, 2* and *3* show the number of transcripts shared by the number of samples they are found in. The graph titled ‘Nucleotides shared’ shows the number of nucleotides transcribed in common in one to seven samples. The *x*-axes correspond to the number of samples sharing a transcript, the *y*-axes show the number of transcripts. The six graphs at the bottom show the number of transcripts per genomic position shared between the samples. Each color represents a different definition: *Definition 1* in gray, *Definition 2* in blue, *Definition 3* in yellow.

The number of identified orthogroups differed between the definitions (Figure 2). *Definition 1* gave the smallest number of orthogroups (9945), *Definition 2* clustered transcripts into 11 305 orthogroups, *Definition 3* into 12 223 orthogroups. *Definition 1* is solely based on transcript similarity and coverage and, contrarily to *Definition 2*, does not take into account the initiation position. Indeed, the fact that *Definition 1* defines fewer orthogroups suggests that around 2000 transcripts overlap but have a substantially different initiation site. *Definition 3* characterizes orthogroups only based on the initiation and termination position of the unspliced transcripts, without considering splicing, similarity or coverage. The fact that this definition gave the highest number of orthogroups, combined with results from *Definitions 1* and *2*, suggests that *de novo* transcripts diverge by their initiation and termination location even though their sequences overlap. In other words, transcripts tend to emerge in nearby genomic regions, which makes them overlap, but at different transcription initiation sites, as they diverge rather in their initiation position than in their coverage. Interestingly, when comparing the number of transcripts shared by samples, the three definitions showed the same frequency spectra (Figure 2). Most orthogroups contain only one *de novo* transcript found in a single sample. The numbers of transcripts shared between samples decrease with the number of samples. This decrease is also visible for each genomic position of transcripts. As *Definition 1* is the least restrictive, it clusters more transcripts from different samples together. Accordingly, the number of transcripts shared by 2–7 samples was higher in *Definition 1* than in *Definitions 2* and *3*. The relative amount of transcripts specific to a single sample represented 53% of the total amount of orthogroups with *Definition 1*, 66% with *Definition 2* and 72% with *Definition 3*. The results from the alternative approach, which counts the number of nucleotides shared between the different samples, cannot be compared quantitatively to the three definitions as transcripts are not considered *per se*. Still, the frequency spectrum shows the same pattern as found by the three definitions.

The same definitions were used to cluster *de novo* transcripts into orthogroups by using *de novo* transcripts whose expression level was larger than 1 TPM (SI, Section A.8 Supplementary Table A2 to A8). The number of orthogroups were expectedly lower but the trend was the same as observed with the expression threshold of 0.5 TPM.

### Gain and loss rates of *de novo* transcripts

We estimated the gain and loss rates for the transcript frequency spectra obtained by *Definitions 2* and *3* (Figure 2). The parameter estimation relies on comparison of a theoretically calculated transcript frequency spectrum and the empirically observed one (details in the Methods section). The estimated parameters are summarized in Table 3, estimated raw parameters for all data sets and models are stated in SI, Section B.7 Supplementary Table B.14.

**Figure 3.**
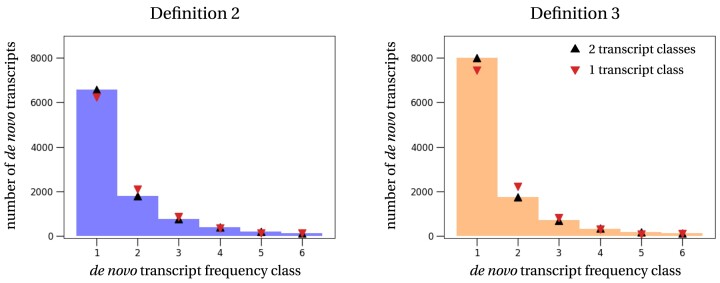
Empirical and estimated transcript frequency spectra. The histograms show the empirical *de novo* transcript frequencies obtained with *Definition 2* (left) and *Definition 3* (right) from the European samples only. Symbols show the theoretical frequency spectrum computed with the parameters estimated by the different models: black triangle – 2 transcript classes; red triangle (upside down) – 1 transcript class.

We explored two different models that differed in the number of transcript classes. In the simpler model, we consider a single class of transcripts, i.e. all transcripts are gained and lost at the same rates. The more complex model distinguishes between two classes of transcripts, a class with a high turnover rate, i.e. fast gain and loss, and a class with a (relatively) lower turnover rate, i.e. slow gain and loss. Overall, the two transcript class model fits the observed frequency distribution slightly better, but the differences are small (Figure 3). We therefore only discuss the estimates from the one transcript class model.

We find high gain and loss rates of transcripts, indicating high (transient) turnover dynamics of *de novo* transcripts. We estimate that between 0.13 and 0.17 new transcripts are gained per year, depending on the definition of transcript orthology and the number of transcript classes. Every single transcript is lost at a rate between 5 × 10^−5^ and 6.3 × 10^−5^ per year, i.e. the expected life span of a transcript is approximately 20,000 years. We note that the gain and loss rates are estimated in rates per generation and need to be transformed to the per year scale (details in the Methods). This parameter transformation strongly depends on the assumptions of the generation time, here assumed to be two weeks (58), and the effective population size of the European *D. melanogaster* population, here set to 900 000 (37). Uncertainty in these parameters can strongly impact the per year rate estimates in Table 3. For example, effective population size estimates range from ∼155 000 (65) to ∼3 100 000 (66).

Lastly, we also estimate the number of transcripts that have become fixed in our data set. By fixed transcripts, we describe transcripts with the following properties: i) They are found in all samples, which means that they have been gained in the most recent common ancestor of the samples, and ii) they are modeled with a loss rate equal to zero. Adding this type of transcript aids the parameter estimation by adding a degree of freedom to better describe the transcripts shared by all samples. We estimate 91–95 fixed transcripts in the one-transcript-class model and 50–52 fixed transcripts in the two-transcript-classes model. We emphasize that the number of fixed transcripts is sample-dependent. Moreover, these transcripts are not necessarily fixed in the species, but may simply be found in all of the samples by chance. Increasing the number of samples will likely reduce the number of estimated fixed transcripts because a newly added sample always has a positive probability of losing one of the transcripts that is shared by all the other samples. For example, by including the Zambian sample, which is an outgroup to the European samples, into the data set, the estimated number of fixed transcripts reduces to 71–75, compared to the estimated 91–95 fixed transcripts within the European samples (Supplementary Table B.9 in the SI).

### Transcript gain and loss rates per genomic region

We estimate the gain and loss rates separately for different genomic regions, using the same methodology as outlined in the previous section. Table 4 shows the estimated parameters (rates per year) for the one-transcript-class model. We find that the loss rates of transcripts are estimated between 2.6 × 10^−5^ and 5.5 × 10^−5^ across all genomic regions, which is consistent with the estimate from the aggregated data (Table 3). The gain rates, in contrast, differ more strongly over the genomic regions. One reason for this large variation might be that the genomic regions do not cover equal proportions of the genome. For example, intergenic regions cover around 68% of the *D. melanogaster* genome, while we approximate that the regions where transcripts overlap with non-coding RNAs only cover around 9% of the genome (details about the estimation of proportions are provided in the SI, Section B.5). Larger genomic coverage should therefore also result in a larger gain rate estimate because there are more positions where a *de novo* transcript could arise. To compare the gain rates across genomic regions in a meaningful way, we therefore normalize them according to their respective coverage. Strikingly, we find that the normalized gain rate of antisense transcripts is almost ten times larger than for all the other regions. Transcript gain in intronic regions is the lowest.

**Table 4.** Estimated *de novo* transcript gain and loss rates per genomic region

	Definition 2				Definition 3			
Genomic region	Gain	Gain_norm_	Loss	*C*_fixed_	Gain	Gain_norm_	Loss	*C*_fixed_
Exon longer	7.7 × 10^−3^	0.05	2.6 × 10^−5^	24	0.01	0.09	3.2 × 10^−5^	31
Intergenic	0.02	0.03	3.9 × 10^−5^	1	0.03	0.05	4.4 × 10^−5^	3
Intronic	3.9 × 10^−3^	0.02	5.5 × 10^−5^	0	8.8 × 10^−3^	0.04	4.5 × 10^−5^	2
ncRNA	3 × 10^−4^	0.04	3.6 × 10^−5^	6	9.1 × 10^−3^	0.1	3.8 × 10^−5^	8
Antisense	0.05	0.35	3.4 × 10^−5^	2	0.07	0.45	4 × 10^−5^	6

We have used the one-transcript-class model to estimate the gain and loss rates of transcripts and the fixed number of transcripts per genomic region. Parameter estimates of gain and loss rates are per year. To compare the gain rates between different genomic regions in a meaningful way, we have normalized the gain rates, denoted by gainnorm, according to the coverage of the region in the genome. Normalization is done by rescaling the gain rates by their respective estimated coverage (details in SI, Section B.6 Supplementary Table B.13).

The estimated parameters from the alternative models and data sets are stated in SI, Supplementary Table B.12. The general pattern remains the same, i.e. we find consistent loss rates across regions, and the lowest gain rate in intronic and the highest gain rate in antisense regions.

## Discussion

### *De novo* transcripts in *Drosophila melanogaster*

We investigated the emergence of *de novo* transcripts by using a unique setup based on samples of *D. melanogaster* from different geographic locations. In each sample, between 2708 and 3116 transcripts with an expression level higher than 0.5 TPM showed no homology to any annotated transcript in Diptera and outgroup species, suggesting their *de novo* emergence (1921 to 2156 with expression level higher than 1 TPM). Some of these detected *de novo* transcripts are the result of alternative splicing, reducing the amount of unspliced *de novo* transcripts to 2327–2809 transcripts per sample. In total, their cumulative length covers 4–6% of the genome. We find that the gain of a transient *de novo* transcript is a frequent event. Previous studies already detected high amounts of new transcripts when comparing species or expression in different organs of the same species. For example, Brown *et al.* (67) identified 1875 new candidate long non-coding RNAs (lncRNAs) producing 3085 transcripts in *D. melanogaster*, with 2990 of them having no overlap with protein-coding genes of *D. melanogaster* or known lncRNAs in outgroup species. Huang et al. (68) determined that 4.5 to 6.7% of transcripts detected in the transcriptome of *D. melanogaster* were not annotated in FlyBase, which amounted to 1669 transcripts derived from intronic regions and 2192 from intergenic regions. We detected fewer *de novo* transcripts in *D. melanogaster* samples than these previous studies. However, the RNA of each of our samples included fewer developmental stages and full body transcripts compared to the other studies, which used tissues from larval, pupal and adult animals. In particular, we detected many fewer transcripts in intronic regions than the two previous studies, which suggests that intronic *de novo* transcripts are specific to some developmental stages or tissues. Moreover, sample-specific genomes were assembled by mapping. Therefore, any transcript arising from a genome rearrangement may have been lost in our study. However, such an event is unlikely as *de novo* genomes did not show major rearrangements (35). Our methodology allowed us to remove all transcripts that correspond to annotated genes from our dataset, confirming that they do not encode for *Drosophila*-annotated proteins. Moreover, we removed any transcript that corresponded to annotated lncRNA from any *Drosophila* species or to transposable elements. However, the data repositories of lncRNAs are likely incomplete, due to their highly variable expression in cells, tissues or individuals. We therefore cannot rule out that some *de novo* transcripts correspond to not yet annotated lncRNAs. Still, as the transcriptomes of the seven samples were extracted under the same conditions in a tightly controlled setting, the lack of expression of *de novo* transcripts in some samples suggests the absence of an established lncRNA, and thus indeed the *de novo* emergence of these transcripts.

Strikingly, we find that in all samples most *de novo* transcripts (around 60%) overlap with coding genes in opposite direction. These antisense transcripts are a common phenomenon in genomes (69). Their functions and mechanisms of emergence were reviewed in Barman *et al.* (70). For example, antisense transcription plays an important role in gene expression regulation, as antisense transcripts can hybridize with forward transcripts and prevent translation of the forward-transcribed transcript (71). Despite their importance in gene regulation, *de novo* emergence of antisense transcripts has not yet been intensively studied. Our results suggest that *de novo* emergence of antisense transcripts is common.

### High estimated rates of gain and loss suggest high transcript turnover

To understand and quantify the transient dynamics of *de novo* transcripts, we rely on the transcript frequency spectrum (Figure 3), i.e. the numbers of transcripts shared between samples. The clustering of *de novo* transcripts into orthogroups forms the basis of this frequency spectrum. We distributed transcripts into orthogroups according to three new definitions that are based on different interpretations of orthology. All definitions resulted in similar patterns of transcripts shared across samples (Fig 2). Strikingly, most *de novo* transcripts were specific to a single sample. This indicates a high transcript gain rate, which in turn suggests a high turnover of transcripts. We find this high rate of transcript gain to be independent of the specific TPM cutoff (main text: 0.5 TPM; SI, Section B.4 Supplementary Tables B9 to B11: 1 TPM and 5 TPM) and independent of the sequence similarity threshold used for the orthogroup definitions (main text: 70%; SI, Section B.5 Supplementary Table B12: 90%). This result is of major importance because it suggests that initiation of transcription is easily gained within a species, but at different genomic locations in individuals. This observation should be taken into account when comparing transcript gains and losses between species, as the choice of the transcriptome representative of the species will impact the results of the comparison.

We quantified the transient dynamics, i.e. gain and loss rates, of *de novo* transcripts based on sequencing data from seven samples of *D. melanogaster*. We emphasize that most of the transcripts in our data are not fixed in *D. melanogaster*, i.e. we do not find them in all samples. Rather, the presence and absence pattern of transcripts throughout the different samples allows us to study the transient state where transcripts are in the process to fixation or extinction within the species. The term *gain* therefore refers to the emergence of a new transcript, but not to its fixation; similarly the term *loss* refers to the loss of a transcript within a sample, but not necessarily to its overall extinction on the species level. Additionally, we estimate the *number of fixed transcripts* in our sample, which are found in all samples and have an assumed loss rate equal to zero. To quantify the transient dynamics, we used the infinitely many genes model (34), and adapted it to the notion of transcripts. One potential limitation of this model is that it does not account for gene flow between samples. However, using an extension of the infinitely many genes model that accounts for horizontal gene transfer (72), the rate of gene flow in our data set is estimated to be zero (details in SI, Section B.8 Supplementary Table B.15). In the context of bacteria, yet another model had been proposed, which estimates gain and loss rates using a fixed phylogenetic tree instead of a coalescent, and which assumes that genes can be gained from an unobserved population or the environment (73). While this might be reasonable for bacteria, which can incorporate genes from the environment, we assume that in our case explicitly accounting for population structure based on the geographic proximity of the samples would be more sensible.

Independent of the definition, we find a high turnover rate of transcripts, i.e. high gain and loss rates. Using the data from *Definitions 2* and *3*, approximately 0.15 transcripts are gained per year. A transcript is lost at a rate of ∼10^−5^−10^−4^ per year, which is consistent across all investigated data sets.

We find fewer orthogroups with *Definition 2* than with *Definition 3*, suggesting that new transcripts diverge rather in their initiation and termination sites than in their coverage. The high turnover of transcripts could be influenced by mutations in transcription factor motifs, strengthening or decreasing their ability to bind to the transcription machinery. This would be consistent with findings in previous studies (9,74,75). For example, high turnover of initiation sites for transcription has been observed between human and mice (76), together with high mutation or rearrangements in upstream regulatory regions (77–84). In *Bacillus subtilis*, 174 transcripts were found to have a new termination signal in the genome, further down than the original termination site, which had been inactivated (85). In fact, several studies in yeast and other eukaryotes demonstrated that modifications in transcription termination were involved in the abundant production of non-genic transcripts (86–88). To maintain constant transcript numbers over time, the large gain rate of transcription is countered by different transcript removal mechanisms. These mechanisms can be complementary and occur at different levels (9,89,90). For example, *de novo* transcripts can be directly degraded in the nucleus or the cytoplasm, as has been shown in *Saccharomyces cerevisiae* (91–93). In addition, transposable elements alter the landscape of pervasive transcription by pausing or terminating neighbouring transcription (94).

### Rates of *de novo* transcript gain vary across genomic regions

We classified *de novo* transcripts according to their genomic position. The proportion of transcripts found per genomic region was similar for all samples (Table 2). The loss rate of *de novo* transcripts was consistent across all genomic regions, which suggests that random mutations are driving the loss of transcription. This is further corroborated by the similar loss rate found in the data sets with the 1 TPM and 5 TPM thresholds. In contrast, we found large differences between the gain rates in the different genomic regions (Table 4). The normalized gain rate was highest for *de novo* transcripts overlapping with genes in the opposite direction of their transcription (antisense). This finding is in line with studies showing that antisense transcription is frequent and is a major driver of evolution as it regulates gene expression (69,95). Moreover, antisense transcripts can also be involved in diseases (70), and their frequent gain and loss could be adaptive in samples that were collected from different geographic locations, and thus possibly different environments. We also consistently find that the rate of transcript gain in intronic regions is lowest. Gain of a new intronic transcript might require both the acquisition of an initiation and a termination site inside an intron, contrarily to the other transcript types, which can potentially exploit an already existing site at one of the two ends. Additionally, the gain of these two elements has to occur in a region limited in size (the intron), which probably explains why gain of transcription inside an intron is less expected.

More unexpectedly, the gain of transcripts in intergenic regions and of transcripts overlapping with a gene occurs at a similar rate. We would have expected that transcripts emerging in overlap with a coding region have a higher chance to be quickly removed by purifying selection (96). Transcripts overlapping with existing genes could, however, have a higher chance to acquire a coding function and by that become part of the gene splicing process, which might also explain the relatively high number of estimated fixed transcripts in this genomic region. These two counteracting processes, purifying selection versus potentially beneficial coding function, could explain why the overall transcript gain rate in regions overlapping with genes in sense is comparable to the suspected selectively neutral dynamics in intergenic regions.

### Conclusion and future prospects

To summarize, we have estimated the transient dynamics of *de novo* transcripts in *D. melanogaster*. These estimates show that *de novo* transcripts are gained and lost at high rates inside this species. Gain rates vary across the genome, being highest in regions overlapping with genes in antisense and lowest in intronic regions. In contrast, loss rates of *de novo* transcripts are found to be similar across the genome.

Larger data sets will help to refine or confirm the generality of our findings. Including more samples, possibly from the same geographic regions or even the same generated isolines, would shed more light on the randomness and transience of *de novo* transcript gain and loss. Moreover, including genomes from several species that follow a phylogenetic gradient would enable modeling transcript dynamics beyond species boundaries. The time of divergence between species would provide insight into the fixation rates of transcripts in species under changing environmental conditions and after going through population bottlenecks. Finally, with the continuous implementation of RNA data bases and whole genome sequencing, further studies could increase the number of samples and by that the comparisons to detect *de novo* transcripts.

In the broader context of genome evolution, our results are a first step to a more mechanistic and less phenomenological treatment and understanding of *de novo* transcript, and consequently *de novo* gene, evolutionary dynamics.

## Supplementary Material

gkad1079_Supplemental_File

## Data Availability

The genomic DNA and RNA sequences are available under NCBI Bioproject PRJNA929424. Additionally, the files containing processed data is available in the Zenodo archive https://doi.org/10.5281/zenodo.7681079, and is referred in the main text as ‘Supplemental Deposit’. The archive contains, for each sample, the polymorphic genomes, transcriptome assemblies, *de novo* transcripts, orthology comparison results, orthogroups according to the 4 classifications, and protein alignments for the phylogenetic reconstruction of the samples in the Supplementary Information. Supplemental figures, information, analyses and models are found in the Supplementary Information. All programs are stored in Zenodo at https://doi.org/10.5281/zenodo.7681078.
